# A case of food protein-induced enterocolitis syndrome to mushrooms challenging currently used diagnostic criteria

**DOI:** 10.1186/2045-7022-5-S3-P162

**Published:** 2015-03-30

**Authors:** Séverine Serafini, Marcel Bergmann, Philippe Eigenmann, Jean-Christoph Caubet

**Affiliations:** 1 Department of Child and Adolescent, Pediatric Allergy Unit, University Hospitals of Geneva, Geneva, Switzerland

## 

We present a case report of food protein-induced enterocolitis (FPIES) to mushrooms and highlight the caveats of diagnosing acute FPIES in clinical practice, particularly in older children and when solid foods are involved. The current clinical diagnostic criteria need to be revised based on the recent evidence to avoid under-diagnosis of FPIES.

